# Oscillatory activity underlying cognitive performance in children and adolescents with autism: a systematic review

**DOI:** 10.3389/fnhum.2024.1320761

**Published:** 2024-02-07

**Authors:** Patricia Soto-Icaza, Patricio Soto-Fernández, Leonie Kausel, Víctor Márquez-Rodríguez, Patricio Carvajal-Paredes, María Paz Martínez-Molina, Alejandra Figueroa-Vargas, Pablo Billeke

**Affiliations:** ^1^Laboratorio de Neurociencia Social y Neuromodulación, Centro de Investigación en Complejidad Social, (neuroCICS), Facultad de Gobierno, Universidad del Desarrollo, Santiago, Chile; ^2^Escuela de Fonoaudiología, Universidad de Valparaíso, Valparaíso, Chile; ^3^Centro de Estudios en Neurociencia Humana y Neuropsicología (CENHN), Facultad de Psicología, Universidad Diego Portales, Santiago, Chile; ^4^Laboratory for Cognitive and Evolutionary Neuroscience (LaNCE), Centro Interdisciplinario de Neurociencia, Facultad de Medicina, Pontificia Universidad Católica de Chile, Santiago, Chile

**Keywords:** autism spectrum disorder, brain oscillations, electrophysiology, review, neurodevelopment, cognitive functions

## Abstract

Autism spectrum disorder (ASD) is a neurodevelopmental condition that exhibits a widely heterogeneous range of social and cognitive symptoms. This feature has challenged a broad comprehension of this neurodevelopmental disorder and therapeutic efforts to address its difficulties. Current therapeutic strategies have focused primarily on treating behavioral symptoms rather than on brain psychophysiology. During the past years, the emergence of non-invasive brain stimulation techniques (NIBS) has opened alternatives to the design of potential combined treatments focused on the neurophysiopathology of neuropsychiatric disorders like ASD. Such interventions require identifying the key brain mechanisms underlying the symptomatology and cognitive features. Evidence has shown alterations in oscillatory features of the neural ensembles associated with cognitive functions in ASD. In this line, we elaborated a systematic revision of the evidence of alterations in brain oscillations that underlie key cognitive processes that have been shown to be affected in ASD during childhood and adolescence, namely, social cognition, attention, working memory, inhibitory control, and cognitive flexibility. This knowledge could contribute to developing therapies based on NIBS to improve these processes in populations with ASD.

## 1 Introduction

Autism Spectrum Disorder (ASD) is a neurodevelopmental condition characterized by two primary behavioral symptomatological dimensions: (i) persistent deficiencies in social communication and interaction across various contexts; and (ii) the presence of restrictive and repetitive patterns of behavior, interests, or activities (American Psychiatric Association, 2013). ASD’s symptomatology extends to a wide range of features, which include atypical sensory-based processing (Orekhova et al., 2008; Marco et al., 2011; Kilroy et al., 2019) and cognitive processing variability, creating a highly heterogeneous profile associated with its multifactorial etiology (Frye et al., 2019; Lord et al., 2020).

From a behavioral perspective, cognitive performance alterations among individuals with ASD have been extensively reported. These alterations encompass deficits in spatial and verbal working memory, set-shifting and response inhibition, among others (Luna et al., 2007; Geurts et al., 2014; Chen et al., 2016; Larrain-Valenzuela et al., 2017; Demetriou et al., 2019, 2018; May and Kana, 2020; Seng et al., 2021). In line with the aforementioned heterogeneity profile in ASD, there is significant interindividual variability in the performance of executive functions within the ASD population (Demetriou et al., 2019; May and Kana, 2020). In particular, the executive function performance in individuals with ASD is argued to depend largely on individual factors such as IQ, age, verbal ability, and general level of cognitive functioning (Luna et al., 2007; Demetriou et al., 2019; May and Kana, 2020). This underscores the complexity of understanding the cognitive features of ASD throughout the lifespan, presenting challenges in comprehending and addressing its diverse manifestations (Luna et al., 2007; Soto-Icaza et al., 2015; Matson, 2017; May and Kana, 2020).

From a neurobiological perspective, altered brain functional stands out as a feature in ASD (Courchesne and Pierce, 2005; Kennedy et al., 2006; Shephard et al., 2019). Several neurobiological mechanisms associated with ASD, spanning molecular to microcircuit alterations, lead to imbalances in excitatory and inhibitory processes in the brain, impacting rhythmogenesis and brain connectivity (Wang et al., 2013). Multiple studies employing different methodologies for measuring brain functioning have reported reduced long-range functional connectivity in ASD (Kikuchi et al., 2015; Kitzbichler et al., 2015; Shephard et al., 2019). One line of research argues that ASD can be characterized by reduced long-range connectivity and increased local connectivity (Courchesne and Pierce, 2005; Happé and Frith, 2006; Peters et al., 2013; Shephard et al., 2019). Another line of findings has also described reduced long-range functional brain connectivity without an increase in local functional connectivity (Kitzbichler et al., 2015). It has been suggested that brain connectivity mediated by slow-frequency oscillations [<25 Hz; (Luck, 2005)] is particularly susceptible to disruption in ASD (Kikuchi et al., 2015; Kitzbichler et al., 2015; Williams et al., 2021). Moreover, atypical sensory processing has also been observed in children (Hudac et al., 2018; Ruiz-Martínez et al., 2020) and in adults (Hudac et al., 2018) with ASD, characterized by heightened responses and slower habituation to auditory stimuli (Hudac et al., 2018; Ruiz-Martínez et al., 2020). This evidence underscores the challenges that individuals with ASD face in effectively processing and encoding sensory information, potentially impacting cognitive performance.

Considering the reported alterations in cognitive performance and brain functioning, which indicate the presence of atypical oscillatory patterns in ASD, this systematic review aims to contribute to the understanding of how these divergent oscillatory patterns are associated with various cognitive domains affected in individuals with ASD. Specifically, we considered studies that investigated brain oscillations in ASD associated with five cognitive domains, namely, social cognition, attention, working memory, inhibitory control, and cognitive flexibility. In this context, three main challenges should be addressed when characterizing specific oscillatory patterns related to cognition functioning: physiological changes associated with the human developmental trajectory and deviations observed in ASD; the variability in populations with ASD, including the presence of comorbidities; and the temporal dynamics between oscillations and specific cognitive processes.

Concerning the developmental trajectory variable, studies on ASD often involve a diverse range of participants, encompassing children, adolescents, and adults. Additionally, research on ASD frequently encompasses individuals with comorbidities and diverse intellectual functioning, individuals with diagnosis of ASD or individuals that have an increased likelihood of having ASD but who do not have a diagnosis, among other factors. To understand the brain mechanisms that impact cognitive processes in ASD, it’s crucial to recognize that cognitive functioning in ASD can be affected independently of comorbidities like intellectual impairments or specific language deficits (Roman-Urrestarazu and Van Kessel, 2022; Zeidan et al., 2022). Consequently, this systematic review specifically focuses on studies involving only children/adolescents with a diagnosis of autism that do not present comorbidities.

Regarding the temporal dynamics between oscillations and specific cognitive processes, brain oscillations can be measured in a task-related manner or during the resting state using different techniques, primarily electroencephalography (EEG) or magnetoencephalography (MEG). The resting state oscillations are relevant because they could reflect a general brain functional architecture, while task-related oscillations could reflect cognitive computations carried out by brain circuits specifically related to the executed task (Mahjoory et al., 2020; Wainio-Theberge et al., 2021). Although both approaches provide valuable information about brain function, we focused on task-related oscillations to obtain a more precise link to the cognitive computations that are altered in ASD that could inform therapeutic interventions. Moreover, since our objective was to review the evidence of alterations in brain oscillations underlying key cognitive processes affected in ASD during childhood and adolescence, we excluded studies that reported Event Related Potentials (ERP) or resting state results. We focused this review on studies that conducted time-frequency analysis to shed light on the oscillatory mechanisms associated with cognitive processes that are affected in ASD (Maguire and Abel, 2013; Nunez et al., 2016).

In the following sections, we outline the methodological steps of this systematic review that revises oscillatory activity associated with specific cognitive functions in children and adolescents with ASD, discuss the behavioral results across different cognitive domains (i.e., social cognition, attention, working memory, inhibitory control, and cognitive flexibility), and summarize neurophysiological findings within specific frequency bands (i.e., theta, alpha, beta, gamma, delta, and mu). Finally, we explore potential implications for understanding the oscillatory brain mechanisms of cognitive functioning in ASD and its relevance for developing therapies based on Non-invasive Brain Stimulation (NIBS).

## 2 Methodology

This scoping review summarizes the oscillatory patterns associated with behavioral performance in different cognitive domains in children and adolescents with ASD. The search of articles was conducted within the framework of behavioral and electrophysiological relations between ASD and no-ASD. The search of experimental articles was made between November and December of 2022, and the studies were extracted from Medline, Scopus, and Web of Science databases without year and language limits. The terms used for the search are described in Table 1.

**TABLE 1:** Searched terms.

Topic	Controlled vocabulary	Natural vocabulary
Population	(”Child”[Mesh] OR “Child, Preschool”[Mesh] OR “Adolescent”[Mesh]) AND (”Autism Spectrum Disorder”[Mesh] OR “Autistic Disorder”[Mesh])	(”Child” OR “Adolescent”) AND (”Autism Disorder” OR “Autism” OR “ASD”)
Area of interest	”Learning”[Mesh] OR “Reversal Learning”[Mesh] OR “Spatial Learning”[Mesh] OR “Verbal Learning”[Mesh] OR “Social Learning”[Mesh] OR “Memory, Short-Term”[Mesh] OR “Social Cognition”[Mesh] OR “Inhibition, Psychological”[Mesh] OR “Mentalization”[Mesh] OR “Theory of Mind”[Mesh]	”Reversal Learning” OR “Learning” OR “Inhibitory Control” OR “Cognitive Flexibility” OR “Working Memory” OR “social cognition” OR “cognitive control”
Outcome	”Brain Waves”[Mesh] OR “Electroencephalography”[Mesh]	”Brain oscillation” OR “Neural Oscillation” OR “neural waves” OR “EEG” OR “frequency band” OR “theta band” OR “gamma band” OR “alpha band” OR “delta band” OR “beta band”

During the screening, all studies were analyzed by title/abstract, full text and one last check during the information extraction process according to PRISMA guidelines (Page et al., 2021). The articles were selected considering the inclusion and exclusion criteria described in Table 2. The inclusion criteria considered that the sample’s age should not exceed 18 years. If the sample encompassed both child and adult populations, it was imperative to ensure that the results were segregated and reported independently for each respective group in order to avoid mixed results. Moreover, studies which considered comorbidity like neurological disorder, congenital conditions, and/or genetic disease and those that did not include a control group were excluded. All the article’ screening was made by seven researchers, and the final data extraction was made by two authors.

**TABLE 2:** Inclusion and exclusion criteria.

Inclusion criteria	The studies must compare children/adolescents with autism and without autism. The outcome should have behavioral data and electrophysiological results recorded with EEG or MEG and should be related to brain oscillations.
Exclusion criteria	Studies which include only adults with autism, children/adolescents with comorbidity and children/adolescents described as having an increased likelihood of autism, but without diagnosis. Research with outcome from EEG or MEG expressed in ERP or Resting State but not in brain oscillations related to a cognitive function.

## 3 Results

**FIGURE 1:**
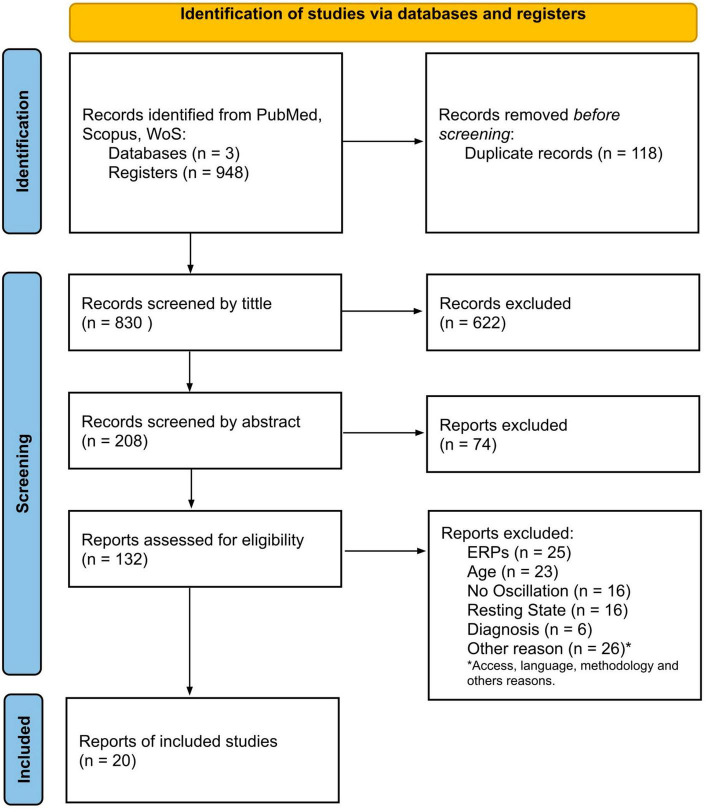
Flow diagram of search and selection.

The article screening was organized according to the PRISMA framework as presented in Figure 1. Next, Table 2 shows the general characteristics of the final article selection. The behavioral data and electrophysiological outcomes are summarized in Tables 3, 4.

**TABLE 3:** Behavioral results.

Cognitive function	Paradigm/task	Behavior results
Social cognition	Join attention (JA) task	There was no difference in the number of JA trials between the two groups. The children with ASD who correctly solved the false belief task were older than children without ASD (Soto-Icaza et al., 2019).
	Action recognition task	The ASD group identified fewer human actions and answered more slowly compared to the NASD group (Sotoodeh et al., 2019).
	Implicit emotional processing task	Neither a significant main effect of emotion nor an interaction effect between emotion and group on response latency was observed (Leung et al., 2014).
Attention	Cue go/NoGo task	The studies used a modified version of the task focusing on an anticipatory cue condition. ASD and NASD groups showed shorter latencies in the cue compared with the no-cue condition that temporally preceded the target stimulus. The average RT was slower for the ASD group than for the NASD group (Beker et al., 2021).
	Kanizsa task	There were no differences in RT between ASD and NASD groups. The ASD group showed more errors in identifying relevant vs. irrelevant stimuli than the NASD group (Casanova et al., 2021).
Working memory	Object recognition (OR) task	The ASD group showed significantly poorer performance than the NASD group on the OR task, showing higher false alarm rates (Chan et al., 2011b).
	N-Back task	The ASD group performed the task less accurately and more slowly than the NASD group (Urbain et al., 2016).
Inhibitory control	go/NoGo task; zoo game	All children showed slower RT for correct answers on Go trials. The ASD group exhibited less accuracy in the taskZoo Game than the NASD group (Buzzell et al., 2022). The ASD group showed significantly more omission errors than the NASD group during the “Go” trials (Chan et al., 2011a).
Cognitive flexibility	WISCONSIN modified version	The ASD group completed fewer blocks, made more perseverative errors, had more failures to maintain sets, and were slower in giving their responses than the NASD group (Yeung et al., 2016).
	Set-shifting paradigm	Children with and without ASD did not differ in their RT in the task (Doesburg et al., 2013).

ASD, autism spectrum disorder; NASD, non-autism spectrum disorder; RT, reaction times; OR, object recognition task; JA, joint attention.

**TABLE 4:** General results.

References	Title	Age group	Sex		Comorbidity	Diagnosis assessment
			ASD group	TD group		
Chan et al., 2011a	Abnormalities in the anterior cingulate cortex associated with attentional and inhibitory control deficits: A neurophysiological study on children with autism spectrum disorders	Child adolescents	20 (1 Girl)	20 (1 Girl)	Asperger syndrome pervasive developmental disorder	DSM-4 CARS Clinical Judgment
Chan et al., 2011b	Disordered connectivity associated with memory deficits in children with autism spectrum disorders	Child adolescents	21 (2 Girls)	21 (7 Girls)	Without comorbidity	DSM-4 CARS Clinical Judgment
Doesburg et al., 2013	Reduced theta connectivity during set-shifting in children with autism	Child	16 (3 Girls)	14 (2 Girls)	Without comorbidity	Not reported
Leung et al., 2014	Reduced beta connectivity during emotional face processing in adolescents with autism	Adolescents	22 (4 Girls)	17 (3 Girls)	Without comorbidity	ADOS-G Clinical Judgment
Jaime et al., 2016	Reduced temporal-central EEG alpha coherence during joint attention perception in adolescents with autism spectrum disorder	Adolescents	16 (2 Girls)	17 (6 Girls)	Without comorbidity	ADOS SCQ
Urbain et al., 2016	Desynchronization of fronto-temporal networks during working memory processing in autism	Child	20 (7 Girls)	17 (4 Girls)	Without comorbidity	ADOS-G Clinical Judgment
Yeung et al., 2016	Abnormal frontal theta oscillations underlie the cognitive flexibility deficits in children with high-functioning autism spectrum disorders.	Child adolescent	25 (6 Girls)	25 (11 Girls)	Without comorbidity	ADI-R
Stavropoulos and Carver, 2018	Oscillatory rhythm of reward: anticipation and processing of rewards in children with and without autism	Child, preschool	20 (1 Girl)	23 (1 Girl)	Without comorbidity	ADOS-2
Soto-Icaza et al., 2019	Beta oscillations precede joint attention and correlate with mentalization in typical development and autism	Child, preschool	20 (7 Girls)	24 (12 Girls)	Without comorbidity	ADOS-2 DSM-5
Sotoodeh et al., 2019	Perception of biological motions is preserved in people with autism spectrum disorder: electrophysiological and behavioral evidences	Child adolescents	20 (3 Girls)	20 (3 Girls)	Without comorbidity	ADI-R DSM-5
Casanova et al., 2021	Ringing decay of gamma oscillations and transcranial magnetic stimulation therapy in autism spectrum disorder	Adolescents	19 (5 Girls)	19 (5 Girls)	Without comorbidity	DSM-4 DSM-5 ADI-R
Beker et al., 2021	Oscillatory entrainment mechanisms and anticipatory predictive processes in children with autism spectrum disorder	Child	31 (7 Girls)	21 (12 Girls)	Without comorbidity	ADOS-2 DSM-5 RBS-R Clinical Judgment
Buzzell et al., 2022	Atypical mediofrontal theta oscillations underlying cognitive control in kindergarteners with autism spectrum disorder	Child, preschool	43 (11 Girls)	24 (10 Girls)	Without comorbidity	ADOS-2

ASD, autism spectrum disorder; TD, typical development; Child, Preschool, A child between the ages of 2 and 5; Child, person 6 to 12 years of age; Adolescents, person 13 to 18 years old; LGDM, likely gene-disrupting mutations; ADHD, attention-deficit/hyperactivity disorder; DSM, diagnostic and statistical manual of mental disorders; ADOS, autism diagnostic observation schedule; DISCO, diagnostic interview for social and communication disorders; ICD-10, international statistical classification of diseases and related health problems; CARS, childhood autism rating scale; ADI-R, autism diagnosis interview, revised; K-ABC, Kaufman assessment battery for children; SCQ, social communication questionnaire; VABS, Vineland adaptive behavior scale-II; RBS, repetitive behaviors scale; K-SADS, kiddie schedule for affective disorders and schizophrenia.

### 3.1 Behavioral results

From the 13 selected studies (Chan et al., 2011a,b; Doesburg et al., 2013; Leung et al., 2014; Jaime et al., 2016; Urbain et al., 2016; Yeung et al., 2016; Stavropoulos and Carver, 2018; Soto-Icaza et al., 2019; Sotoodeh et al., 2019; Beker et al., 2021; Casanova et al., 2021; Buzzell et al., 2022), five articles reported findings from social cognition paradigms (Leung et al., 2014; Jaime et al., 2016; Stavropoulos and Carver, 2018; Soto-Icaza et al., 2019; Sotoodeh et al., 2019), two studies reported findings from attentional tasks (Beker et al., 2021; Casanova et al., 2021), two from working memory paradigms (Chan et al., 2011b; Urbain et al., 2016), two from inhibitory control paradigms (Chan et al., 2011a; Buzzell et al., 2022), and two from cognitive flexibility paradigms (Doesburg et al., 2013; Yeung et al., 2016). It is worth noting that from these 13 articles, three studies did not report accuracy or latency results due to their experimental designs. In one of these studies (Soto-Icaza et al., 2019), the outcome to observe was a spontaneous behavior that depends on the child’s preference without any measure of correct or incorrect performance. In the other study, the experimental paradigm involved a guessing task where the probability of winning did not depend on the participant (Stavropoulos and Carver, 2018). Finally, in the third study, the accuracy measure was assessed in a different time and test context than the task in which the EEG was recorded. This means the accuracy results do not correspond to the EEG findings obtained during the specific task (Jaime et al., 2016).

Regarding studies that evaluated the social cognition domain, only one (Soto-Icaza et al., 2019) of the two studies that assessed joint attention ability (Jaime et al., 2016; Soto-Icaza et al., 2019) reported behavioral results, revealing no differences between the two groups of children in initiating joint attention (Soto-Icaza et al., 2019). Similarly, within an implicit emotional face processing task (Leung et al., 2014), neither a significant main effect of emotion nor an interaction effect between emotion and group on response latency was observed. Nevertheless, a study that assessed an action recognition task (Sotoodeh et al., 2019), reported that the ASD group of children and adolescents answered slower and less correctly identified human actions than the Non-autism spectrum disorder (NASD) group of participants.

Concerning behavioral results in the context of attentional tasks, while one study reported that the average reaction time was slower for children with ASD than for children without ASD (Beker et al., 2021), another study did not observe group differences in the reaction time between ASD and NASD groups of children (Casanova et al., 2021). However, it was reported that the group of children with ASD exhibited more errors in identifying the relevant vs. the irrelevant stimulus than the NASD group (Casanova et al., 2021).

Likewise, in the working memory experimental paradigms, a study reported that children with ASD had higher false alarm rates than the NASD group (Chan et al., 2011b), while another study found that children with ASD performed less accurately and more slowly than children without ASD (Urbain et al., 2016).

Results in inhibitory control showed that while children with and without ASD exhibited slower reaction times for correct answers on “Go” trials compared to error responses in “NoGo” trials, the ASD group showed less accuracy for both “Go” and “NoGo” trails than the NASD group (Buzzell et al., 2022). Furthermore, the ASD group exhibited significantly more omission errors than the NASD group during the “Go” trials (Chan et al., 2011a).

Finally, while a study reported no differences between NASD and ASD groups in their reaction times among different conditions in a cognitive flexibility experimental task (Doesburg et al., 2013), children and adolescents with ASD completed fewer blocks, committed more perseverative errors, had a higher rate of failures to maintain sets, and exhibited slower response times compared to the NASD group in a modified version of the Wisconsin test (Yeung et al., 2016).

Overall, a cognitive domain can be assessed with several experimental paradigms. This variability in tasks makes it challenging to find behavioral patterns in ASD. However, a tendency among the studies included in this review showed that children and adolescents with ASD tend to exhibit both a poorer (Chan et al., 2011a,b; Urbain et al., 2016; Sotoodeh et al., 2019; Casanova et al., 2021; Buzzell et al., 2022) and slower performance in experimental tasks (Urbain et al., 2016; Yeung et al., 2016; Sotoodeh et al., 2019; Beker et al., 2021) or no differences (Chan et al., 2011a; Doesburg et al., 2013; Leung et al., 2014; Jaime et al., 2016; Soto-Icaza et al., 2019; Buzzell et al., 2022) when compared to participants without ASD, but never a higher performance when compared to participants without ASD.

### 3.2 Brain oscillations results

In the following subsections, we describe the results related to oscillatory activity, categorized by frequency bands. In the literature, the terms “event-related synchronization” and “event-related desynchronization” are frequently used to refer to increases and decreases in power following a stimulus (Pfurtscheller and Lopes Da Silva, 1999). To prevent confusion related to inter-site synchronization measurement and to ensure clarity when emphasizing distinctions compared to different conditions rather than baseline measures, we use the more descriptive terms “increases” and “decreases” in power.

#### 3.2.1 Theta-band brain oscillations

Cortical theta-band oscillations (frequencies ranging from 4 to 8 Hz) have been proposed as a key mechanism through which neurons can compute and communicate top-down control across extensive networks (Cavanagh and Frank, 2014). Theta brain waves have been widely described in association with high-level cognitive processes, such as memory, cognitive control, performance monitoring, social cognition, and decision-making (Sauseng et al., 2010; Cavanagh and Frank, 2014; Hsieh and Ranganath, 2014; Roux and Uhlhaas, 2014; Tafuro et al., 2019; Herweg et al., 2020; Riddle et al., 2020).

**FIGURE 2:**
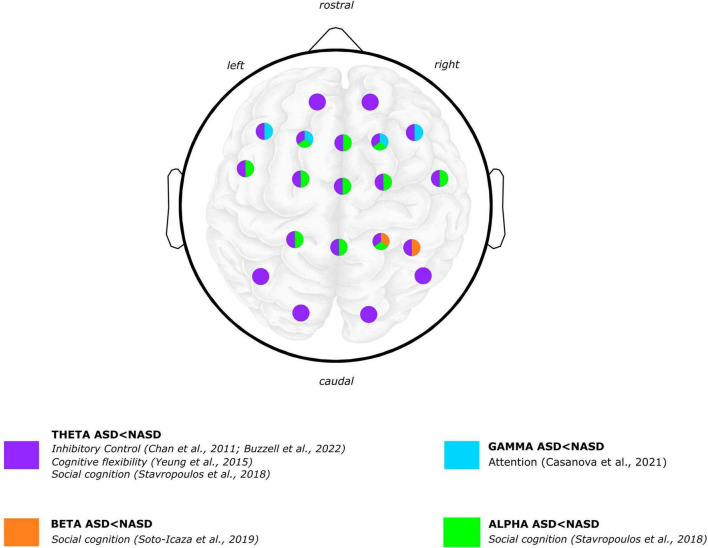
Graphical summary of the differences between ASD and NASD groups. Brain oscillatory results: power frequency findings. EEG scalp and brain image are shown for illustrative purposes only. Colors represent regions where differences were reported in findings between the ASD and NASD groups. Articles Jaime et al. (2016) and Beker et al. (2021) are not shown in the figure because oscillatory topology is not specified in the results. Brain image designed by rawpixel.com/Freepik.

Concerning ASD, findings with inhibitory control tasks coincide in describing that children with ASD exhibit lower theta-band power than children without ASD (Chan et al., 2011a; Buzzell et al., 2022; Figure 2). Specifically, a study showed that children with ASD exhibited lower mediofrontal and late theta power related to errors (Buzzell et al., 2022). Furthermore, compared to children without ASD, children with ASD exhibited a significant decrease in theta power within both the anterior brain region during the “Go” condition and in anterior and centrotemporal regions during the “No-Go” condition (Chan et al., 2011a). In a reward processing task, ASD group exhibited a lower theta-band power irrespective of whether the rewards were social or non-social (Stavropoulos and Carver, 2018).

Furthermore, in a cognitive flexibility paradigm, a study found that the ASD group exhibited lower frontal theta power compared to the NASD group (Yeung et al., 2016). This difference was only observed in a late stage of the task (i.e., 600 ms after the stimulus onset). Posterior theta activity was not different between groups (Yeung et al., 2016).

There is also a study that did not observe any significant power differences between ASD and NASD participants. Using an attentional paradigm in which children had to anticipate an auditory stimulus after visual cues, the study showed no significant power differences between ASD and NASD groups neither in theta nor in delta band power (Beker et al., 2021).

Regarding the studies that used coherence measures, a reduced theta-band network synchronization was observed in children with ASD in a cognitive flexibility task (Doesburg et al., 2013). This reduced theta-band connectivity was exhibited only under the most demanding condition of the task. Additionally, in a working memory task, children with ASD exhibited significantly higher long-range coherences in the left hemisphere. Specifically, compared to children without ASD, children with ASD exhibited higher connections at the left intra-hemispheric and left-to-right inter-hemisphere regions (Chan et al., 2011b).

In summary, findings indicate that children with ASD display lower theta-band power compared to children without ASD in inhibitory control tasks. Moreover, across different tasks such as reward processing and cognitive flexibility, the ASD group exhibits lower theta-band power and reduced theta-band network synchronization. However, studies that used attentional paradigms, do not report significant power differences between ASD and NASD participants. Conversely, in a working memory task, children with ASD exhibit significantly higher long-range coherences, emphasizing the complexity and variability of theta-band dynamics in the neurocognitive profile of individuals with ASD.

#### 3.2.2 Alpha-band oscillations

It has been shown that when recording brain activity from the human scalp, the dominant frequencies are in the alpha and beta ranges, which span from 8 to 12 Hz and 13 to 30 Hz, respectively (Pfurtscheller and Lopes Da Silva, 1999; Kilavik et al., 2013; Piai et al., 2015; Nyhus, 2018; Griffiths et al., 2019; Barone and Rossiter, 2021). Intracranial recordings have demonstrated that alpha oscillations, generated through cortical and thalamocortical mechanisms (Pfurtscheller and Lopes Da Silva, 1999; Weiner et al., 2023), are observable in parietal, visual, motor, and auditory cortices. Synchronized alpha-band oscillations have been observed during mental inactivity, often described as an “idling system” (Pfurtscheller and Lopes Da Silva, 1999; Buzsáki and Draguhn, 2004). Although desynchronization in the alpha-band frequency range, characterized by an amplitude decrease of this frequency is interpreted as cortical activation (Pfurtscheller and Lopes Da Silva, 1999; Buzsáki et al., 2012; Klimesch, 2012; Halgren et al., 2019), different spectral features of alpha rhythm show separable correlations with brain networks related to task execution and rest (Sadaghiani and Kleinschmidt, 2016).

Concerning alpha-band power in cognitive performance in ASD, one study found higher alpha power displayed in adolescents with ASD compared to adolescents without ASD while responding to joint attention (Jaime et al., 2016). Also in a social cognition paradigm, a study reported that children with ASD showed both a lower left alpha power (i.e., suppression or desynchronization) during the anticipation of a non-social reward and a higher left alpha power during the anticipation of a social reward processing compared to children without ASD (Stavropoulos and Carver, 2018). Regarding the processing of social and non-social rewards, children with ASD exhibited more alpha suppression than children without ASD regardless of the reward type (Figure 2).

Regarding alpha connectivity studies, a research of interregional phase synchronization across trials (Urbain et al., 2016) observed a reduced frontotemporal alpha synchronization in ASD compared to children without ASD in a working memory task. Another study measured neuronal entrained as inter-trial phase coherence, where the phase-locking of the alpha brain oscillatory activity to rhythmic sensory inputs was extracted (Beker et al., 2021). Using an anticipatory experimental paradigm in which children had to pay attention to a set of visual cues temporally preceding an auditory target, a reduced alpha-band oscillatory activity was observed in the ASD group (Beker et al., 2021). Finally, a study of synchronized oscillatory activity among different neural nodes (Nunez et al., 2016) reported a significant reduction in temporal–central alpha coherence in adolescents with ASD in a social cognition paradigm (Jaime et al., 2016).

In summary, the evidence related to alpha-band power in cognitive performance among individuals with ASD shows diverse patterns. In working memory tasks, reduced frontotemporal alpha synchronization was observed, and a decrease in alpha-band oscillatory power was found in anticipatory paradigms. Adolescents with ASD demonstrated higher alpha power during joint attention, while social cognition paradigm revealed a significant reduction in alpha coherence among adolescents with ASD. These findings underscore the heterogeneous nature of changes in alpha-band oscillations in neurocognitive processes in ASD.

#### 3.2.3 Beta-band brain oscillations

Beta-band oscillations (ranging from 13 to 30 Hz) have been widely described as being associated with voluntary movement that induces a specific desynchronization followed by a post-movement synchronization of this oscillatory band (Pfurtscheller and Lopes Da Silva, 1999; Griffiths et al., 2019). Additionally, studies in cognitive processing have highlighted the involvement of beta oscillation in cortical connectivity in several processes that require top-down control, such as memory processing (Griffiths et al., 2019; Forner-Phillips et al., 2020; Herweg et al., 2020), focused attention (Buschman and Miller, 2007; Buschman et al., 2012; Richter et al., 2017), and performance monitoring (Billeke et al., 2020), among others.

Regarding ASD, children with ASD exhibited a smaller beta-band power in the right parietal region during a social experimental paradigm of initiating joint attention compared to children without ASD (Soto-Icaza et al., 2019). Also in a joint attention paradigm, adolescents with ASD demonstrated inverse laterality of beta band power (greater in left hemisphere) compared to NASD group, with no difference in the overall power between groups (Jaime et al., 2016).

Connectivity analysis has shown inconclusive results (Leung et al., 2014; Jaime et al., 2016). While one study found no differences between groups during joint attention (Jaime et al., 2016), a reduced beta-band phase-locking in adolescents with ASD was associated with a face emotional recognition task in another study (Leung et al., 2014).

In summary, children with ASD displayed lower beta-band power in right parietal regions during a joint attention task, while adolescents exhibited the inverse phenomenon, greater power in the left hemisphere. Connectivity analyses provided inconclusive results. These findings underscore how beta-band dynamics seem to vary during development in individuals with ASD during social interactions.

#### 3.2.4 Gamma-band brain oscillations

The frequency range of gamma oscillations has been characterized as fluctuating between 30 to 90 Hz (Başar et al., 2015). Although several studies have reported gamma oscillations related to diverse cognitive functions (Herrmann et al., 2004; Başar et al., 2015) and top-down integration (Pfurtscheller and Lopes Da Silva, 1999; Buschman and Miller, 2007; Richter et al., 2017; Casanova et al., 2021), scalp recording faces several technical difficulties that challenge a straightforward interpretation of the results (Herrmann et al., 2004; Başar et al., 2015).

Regarding ASD, one study that observed gamma oscillations associated with the attentional processing of relevant vs. irrelevant stimuli reported that children and adolescents with ASD exhibited significant differences in “evoked” gamma oscillations (Casanova et al., 2021). “Evoked” gamma refers to oscillations that are phase-locked to stimulus presentation. It has been argued that the early (“evoked”) gamma oscillation could be considered an attention–triggering process and that late (“induced”) gamma responses reflect a more detailed processing of the stimulus (Casanova et al., 2021, 2020). Specifically, regarding “evoked gamma,” the ASD group showed a lower peak amplitude of responses to target than non-target and irrelevant stimuli. In contrast, the NASD group of participants exhibited higher gamma amplitude to target than non-target and irrelevant stimuli (Figure 2).

In summary, the investigation of gamma oscillations associated with attentional processing revealed significant differences in “evoked” gamma oscillations in children and adolescents with ASD when compared to children and adolescents without ASD. This suggests distinct patterns in attention-triggering processes between individuals with and without ASD, emphasizing the intricate dynamics of gamma oscillations in the context of attentional processing in ASD.

#### 3.2.5 Mu-band brain oscillations

The mu-band oscillations (also known as “upper alpha,”) (Pfurtscheller and Lopes Da Silva, 1999) are brain waves between the 8 to 13 Hz range specifically associated with the sensorimotor cortex (Pfurtscheller and Lopes Da Silva, 1999; Démas et al., 2020; Chen et al., 2021). When an individual either performs, observes, or even imagines a movement, there is a noticeable attenuation in the power of mu oscillations, a phenomenon known as “mu band suppression” (Démas et al., 2020; Chen et al., 2021). The mu band suppression exhibits sensitivity to animated objects and goal-directed actions (Raymaekers et al., 2009), being associated with social tasks as neural correlates of the “mirror neuron system” (Oberman et al., 2005; Dapretto et al., 2006; Cattaneo and Rizzolatti, 2009; Raymaekers et al., 2009).

Within the context of ASD, evidence has shown inconclusive results (Oberman et al., 2005; Dapretto et al., 2006; Cattaneo and Rizzolatti, 2009; Raymaekers et al., 2009; Sotoodeh et al., 2019). Only one study that reported mu met our inclusion criteria (Sotoodeh et al., 2019), showing no significant statistical differences between a group of children and adolescents with and without ASD in identifying human movements using a point light. Also, none of both groups presented a correlation of age or intelligence quotient (IQ) with mu-band suppression.

In summary, evidence of mu oscillations yields inconclusive results. The limited available evidence related to this frequency band underscores the need for further research to elucidate the role of mu oscillations in ASD and its potential correlation with cognitive and developmental issues that characterize this disorder.

## 4 Discussion

Here we summarize the oscillatory electrophysiological evidence on cognitive functioning in children and adolescents with ASD. Behavioral evidence has systematically shown a broad executive dysfunction in ASD (Demetriou et al., 2018). Consistent with these findings, our results also reveal that children and adolescents with ASD tend to exhibit either poorer and/or slower performance in experimental tasks or show no significant differences when compared to participants without ASD. There is no included study in which the ASD group exhibits higher performance levels when compared to participants without ASD. This behavioral trend suggests a common thread of cognitive dysfunction in ASD across the revised experimental paradigms, highlighting the consistency of impairment across various cognitive domains.

When examining brain oscillatory functioning that underlies cognitive performance in children and adolescents with ASD, results can be disaggregated into power analysis and functional connectivity studies. Irrespective of the frequency band, the connectivity analysis or the cognitive domain, most of the studies evidenced differences between ASD and NASD groups. As with behavioral findings, this oscillatory trend is in line with the evidence that has described an atypical brain functioning in ASD. Considering that ASD has been related to an imbalance in excitatory and inhibitory brain activity (Rubenstein and Merzenich, 2003; Fatemi et al., 2009; Yizhar et al., 2011; Zikopoulos and Barbas, 2013; Weder et al., 2014; Boes et al., 2018; Zhao et al., 2022; Valdebenito-Oyarzo et al., 2024), and that functional connectivity has been related to a mediation of slow frequency oscillations (<25 Hz) (Pfurtscheller and Lopes Da Silva, 1999; Luck, 2005; Buschman and Miller, 2007; Griffiths et al., 2019; Forner-Phillips et al., 2020), the role of low oscillations in timing of neuronal activation processes becomes essential when trying to better understand ASD. Brain oscillations interact across various frequency bands, influencing one another and orchestrating specific behaviors (Pfurtscheller and Lopes Da Silva, 1999; Buzsáki and Draguhn, 2004; Buschman and Miller, 2007; Jensen and Mazaheri, 2010; Klimesch, 2012). Scalp recordings exhibit synchronized and desynchronized activity across different frequency bands occurring at the same moment in time (Pfurtscheller and Lopes Da Silva, 1999; Griffiths et al., 2019; Forner-Phillips et al., 2020). The overall trend observed in our review in which slower frequency bands exhibit lower power in children and adolescents with ASD, could be reflecting the excitatory/inhibitory imbalance that is closely related to cognitive difficulties. Evidence has shown that slow oscillations encompass larger synchronous membrane voltage fluctuations across broader brain regions than fast oscillations (Pfurtscheller and Lopes Da Silva, 1999; Jensen and Mazaheri, 2010; Klimesch, 2012; Buskila et al., 2019). Considering the evidence of disruptions in neuroanatomical and cell organization in the ASD brain as evidenced by postmortem and structural neuroimaging studies (Jensen and Mazaheri, 2010; Chen et al., 2016; Buskila et al., 2019; Sokoliuk et al., 2019; Cellier et al., 2021), and regarding slower oscillations as brain pacemakers (Pfurtscheller and Lopes Da Silva, 1999; Jensen and Mazaheri, 2010; Klimesch, 2012; Buskila et al., 2019), it is possible to interpret that disturbances in the mechanisms regulating excitation or inhibition of sensory-specific regions might affect the facilitation or filtering of sensory processing when needed (Buzsáki and Draguhn, 2004; Jensen and Mazaheri, 2010; Klimesch, 2012; Sokoliuk et al., 2019). Connectivity differences between ASD and NASD groups also observed in this review could also be related with this atypical brain functioning. The ability to prioritize important target-relevant information while filtering out irrelevant data is crucial when it comes to cognitive performance. Indeed, evidence has described how this complex interplay between focusing and filtering -between prioritizing and suppressing-, can be achieved by the coupling of frequencies among brain regions (Cavanagh and Frank, 2014; Riddle et al., 2020).

As previously mentioned, understanding the mechanisms underlying brain functioning in individuals with ASD is challenging due to the complexity of its symptoms and etiology. Furthermore, comprehending the neurodevelopment of these mechanisms requires considering additional variables. The development of cerebral oscillations during childhood and adolescence plays a crucial role in understanding the atypical neural oscillatory trajectory that is present in this neurodevelopmental disorder (Cellier et al., 2021). Unlike the evidence described in adults (Klimesch, 2012; Griffiths et al., 2019), it has been found that during early childhood, theta oscillations in posterior electrodes is the predominant oscillation in the brain (Cellier et al., 2021). It has also been observed that the peak frequency of the dominant oscillation in the alpha range increases between the ages of 7 and 24 (Cellier et al., 2021). Additionally, it has been shown that the distribution of theta oscillations transitions from being dominant in the posterior region of the scalp during early childhood to anterior electrodes in adulthood (Cellier et al., 2021). Interestingly, evidence in spontaneous brain rhythms show a shift toward higher frequencies with age (Rodríguez-Martínez et al., 2017). Specifically, significant differences in absolute spectral power are observed across all EEG frequencies (excluding the gamma rhythm) from childhood to young adulthood (Rodríguez-Martínez et al., 2017). This kind of evidence points out the importance of including sample age specifications that make it possible to address these constraints.

Several methodological biases and limitations are important to consider. First, studies typically emphasize “positive” findings, focusing on what has been found rather than explicitly describing what has not been observed. In this systematic review, only one study (Beker et al., 2021) explicitly acknowledged a deviation in the obtained oscillatory result from the anticipated outcome. Unanticipated “negative” findings can be tremendously informative, especially when considering the complexity of neurodevelopmental conditions as heterogeneous as ASD.

Second, it is crucial to mention the sex difference in the experimental samples of the studies. Epidemiologically, ASD has been characterized as a disorder with a higher prevalence in men than in women (Dapretto et al., 2006; American Psychiatric Association, 2013). There is broad agreement that the men: women gender ratio in ASD is approximately 4:1 (Oberman et al., 2005; Roman-Urrestarazu and Van Kessel, 2022; Zeidan et al., 2022; Zhao et al., 2022). However, this statistical calculation has started to be revised and questioned thanks to clinical studies that have emphasized the existence of an apparent gender bias in the diagnosis. In this regard, women who meet the criteria for ASD are disproportionately at risk of not receiving a clinical diagnosis (Rubenstein and Merzenich, 2003; Oberman et al., 2005; Fatemi et al., 2009; Yizhar et al., 2011; Zikopoulos and Barbas, 2013; Weder et al., 2014). It is now known that, of children who meet the criteria for ASD, the actual man-to-woman ratio is closer to a 3:1 ratio rather than the 4:1 ratio (Oberman et al., 2005). This sex bias is also evidenced in this revision. While all of the selected studies incorporate both girls and boys in their research samples, a common issue across all of them is the imbalance in gender representation. In fact, two of the 13 selected studies consider only one girl in a sample of 20 participants with ASD (Chan et al., 2011a; Stavropoulos and Carver, 2018), and six of them consider five or less girls in samples ranging from 16 to 22 participants with ASD (Chan et al., 2011b; Doesburg et al., 2013; Leung et al., 2014; Jaime et al., 2016; Sotoodeh et al., 2019; Casanova et al., 2021). While it is true that this sample unbalance could be a reflection of the epidemiological ratio, it is also true that it leads us to results that are biased due to the underrepresented features of ASD in girls which are crucial for a proper diagnosis.

It is also important to state the limitations of this review. Considering that it has been shown that there are alterations in oscillatory features associated with cognitive functions in ASD, our main objective was to study the brain oscillations that underlie key cognitive processes that have been shown to be affected in ASD. The inherent constraints of using criteria focused solely on task-related brain oscillations entails the exclusion of spontaneous (non-task related) brain oscillations (Rodríguez-Martínez et al., 2017). It has been reported that the dynamics of diverse electrophysiological variables reveal distinct relationships between their spontaneous and evoked activities (Wainio-Theberge et al., 2021). Interestingly, a recent meta-analysis showed that when analyzing resting state brain activity, individuals with ASD exhibit reduced relative alpha power and increased gamma power, while delta, theta, and beta power remain similar to those without ASD. These findings underscore the importance of exploring resting-state alpha and gamma power as potential biomarkers for autism, fostering further investigation in this direction (Neo et al., 2023).

In summary, the evidence presented here reveals distinct oscillatory patterns associated with cognitive deficits in ASD. While further research is required to establish precise connections between oscillatory brain activity and specific cognitive impairments in this neurodevelopmental disorder, the findings underscore alterations in spectral features in ASD. These findings have implications for understanding the neural basis of cognitive impairments, which may inform potential therapeutic interventions. Recent technological advancements have enabled the development of non-invasive brain stimulation therapies for neurological and psychiatric disorders (Boes et al., 2018). NIBS techniques such as Transcranial Magnetic Stimulation (TMS) or transcranial alternating current stimulation (tACS) can be tailored to modulate both specific cognitive processes (Valdebenito-Oyarzo et al., 2024) and their underlying brain oscillations (Beliaeva et al., 2021). For instance, tACS applies sinusoidal currents to the intact scalp of individuals to directly modulate ongoing brain oscillations (Johnson et al., 2020). Recently, this technique has been used in clinical settings to treat symptomatology in neuropsychiatric diseases (Elyamany et al., 2021). While promising evidence indicates the potential of these interventions to improve specific cognitive processing (Grover et al., 2022), there is still a lack of evaluation of these effects using randomized clinical trials (Elyamany et al., 2021). Additionally, the variability observed in the results of tACS applications is attributed to the diverse protocols employed, including variations in frequency range and electrode position. In this context, systematic reviews that establish connections between specific cognitive computations and oscillatory activity in brain networks, such as the one that we present here, can offer valuable insights for designing rational interventions based on NIBS that can be used to treat neuropsychiatric conditions. Furthermore, these kinds of reviews can also help to identify evidence gaps that may be addressed in new research on the electrophysiological functioning of the brain in neurodevelopmental conditions such as ASD.

## Data availability statement

The raw data supporting the conclusions of this article will be made available by the authors, without undue reservation.

## Author contributions

PS-I: Conceptualization, Formal Analysis, Funding acquisition, Methodology, Project administration, Supervision, Visualization, Writing – original draft, Writing – review and editing. PS-F: Conceptualization, Formal Analysis, Methodology, Project administration, Supervision, Visualization, Writing – original draft, Writing – review and editing. LK: Conceptualization, Formal Analysis, Writing – original draft, Writing – review and editing. VM-R: Formal Analysis, Writing – review and editing. PC-P: Formal Analysis, Writing – review and editing. MM-M: Formal Analysis, Writing – review and editing. AF-V: Formal Analysis, Writing – review and editing. PB: Conceptualization, Formal Analysis, Funding acquisition, Supervision, Visualization, Writing – original draft, Writing – review and editing.
